# Macroscopic to Microscopic – A Case of Crohn’s Disease Progressing to Collagenous Colitis

**DOI:** 10.7759/cureus.18299

**Published:** 2021-09-26

**Authors:** Murtaza Shabbir Hussain, Harika Balagoni, Sankalp Dwivedi, Marc Piper

**Affiliations:** 1 Internal Medicine, Hurley Medical Center, Flint, USA; 2 Gastroenterology, Ascension Providence Hospital, Southfield, USA

**Keywords:** microscopic colitis, inflammatory bowel disease, collagenous colitis, lymphocytic colitis, gi endoscopy

## Abstract

The association between microscopic colitis (MC) and inflammatory bowel disease (IBD) is uncertain and infrequently reported. Rare cases in the literature consist of simultaneous MC and IBD, or progression of one condition to the other. We present a unique case of clinically and endoscopically diagnosed and successfully treated IBD that revealed MC on histology months later due to reappearance of diarrhea. Common pathophysiologic mechanisms, such as tumor necrosis factor α and T helper type 1 cells, may explain the MC and IBD relationship. During endoscopy, a prompt biopsy should be taken if suspicious for MC, thus decreasing the duration of patient's symptoms and saving healthcare costs.

## Introduction

In resource-poor countries, symptoms of diarrhea are one of the top leading causes of death due to severe dehydration, regardless of the cause. On the other hand, in resource-rich countries, diarrhea is more of an ‘inconvenience’ presenting as a common concern in ambulatory care. Majority of cases are infectious and of viral etiology. Noninfectious causes include malabsorption disorders, functional disorders, and organic disorders such as inflammatory bowel disease (IBD) and microscopic colitis (MC). MC is divided into collagenous colitis (CC) and lymphocytic colitis (LC) and presents as chronic, watery diarrhea but normal-appearing mucosa on colonoscopy [1,2]. IBD, classified into ulcerative colitis (UC) and Crohn’s disease (CD), is an autoimmune condition that clinically presents with abdominal pain, bloody or non-bloody diarrhea, weight loss, and evidence of inflammation on endoscopy. IBD and MC are distinct regarding their epidemiology, clinical manifestations, management, and pathology. There is a rare incidence of MC in known IBD patients and vice versa [3]. Equally as rare is the concurrent manifestation of IBD and MC.

We report a patient who was treated for clinically diagnosed CD. The recurrence of diarrheal symptoms resulted in further testing, which led to the diagnosis of CC.

## Case presentation

**Figure 1:**
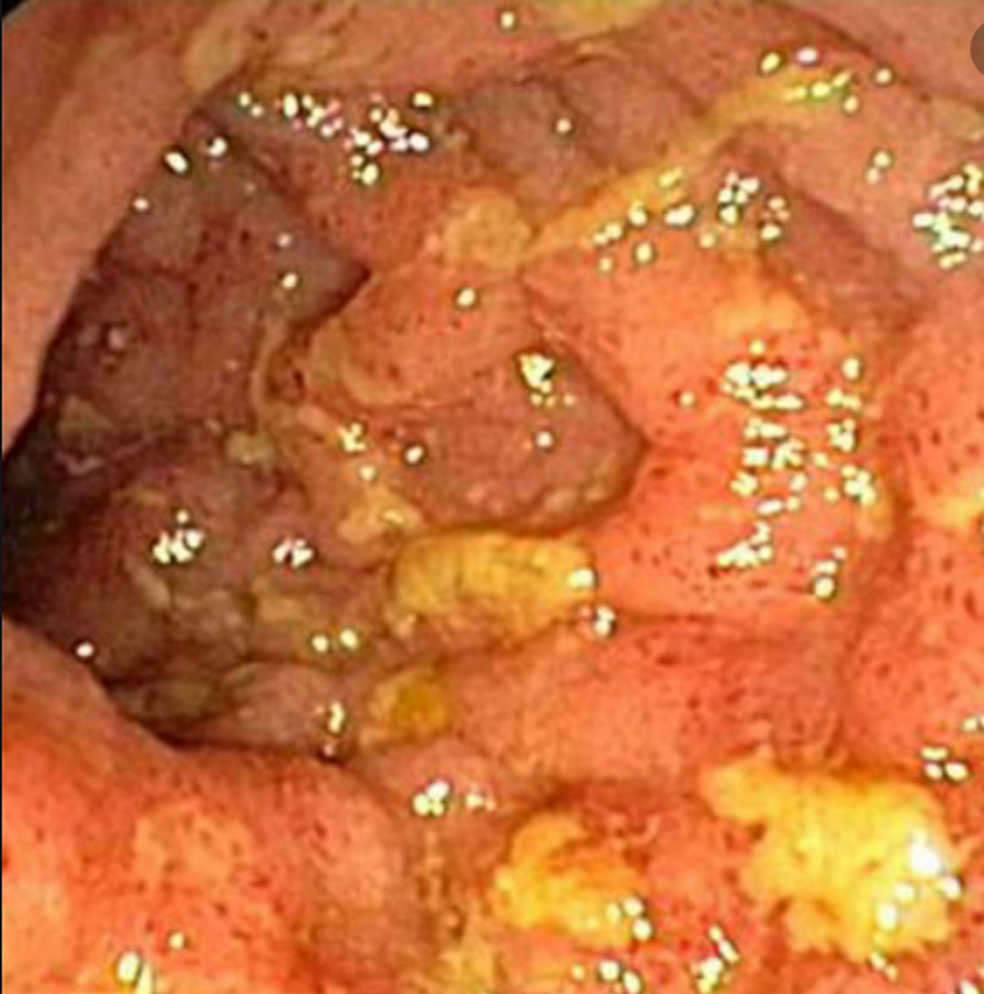
Initial colonoscopy demonstrating terminal ileitis

**Figure 2:**
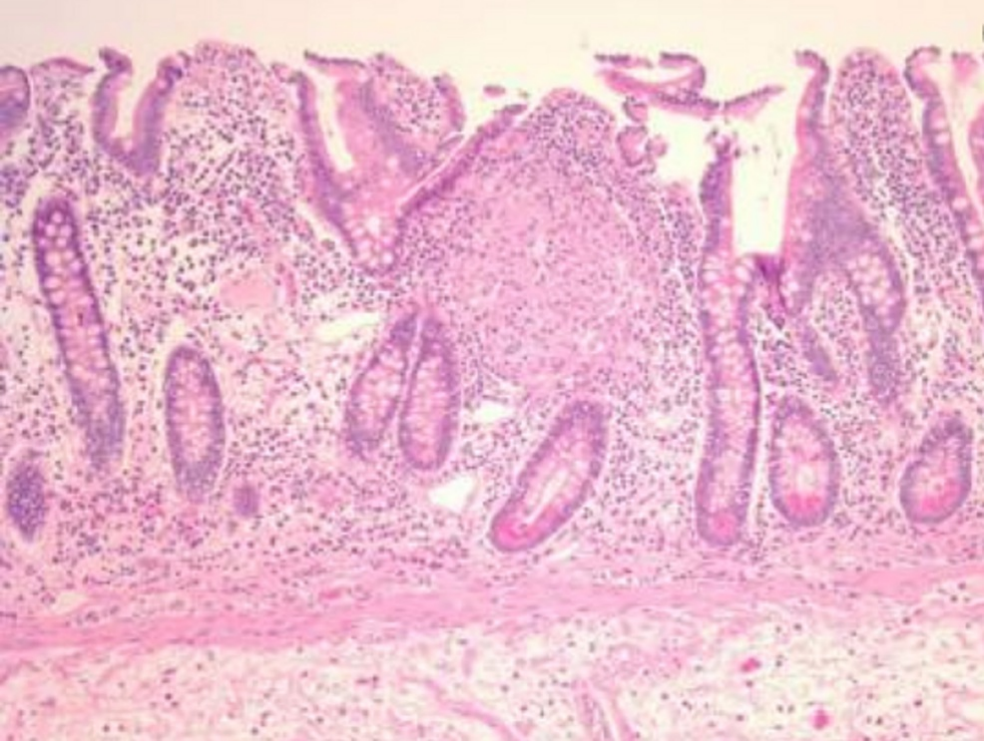
Hematoxylin and eosin stain biopsy reveals mild-chronic active colitis without granulomas or dysplasia

A 56-year-old-female with a history of anxiety and chronic smoking presented to our hospital with 12-day progressive, non-bloody diarrhea, 20-30 episodes/day including nocturnal episodes, associated with diffuse, cramping, lower abdominal pain. She denied fever, chills, sick contacts, travel, food contamination, medication changes, or recent antibiotics. She tried Imodium at home with no improvement. On presentation, she had mild leukocytosis, mild transaminitis, elevated C-reactive protein of 102 mg/L and fecal calprotectin 484 mcg/g. Computed tomography (CT) abdomen/pelvis did not show any acute abnormalities. Stool studies were negative for infection. She underwent esophagogastroduodenoscopy, which showed mild gastritis and duodenitis, biopsies negative for *Helicobacter pylori* and celiac disease. Colonoscopy showed mild to moderate colitis with mucosal congestion, granularity, and cobble-stoning in a continuous and circumferential pattern from the rectum to the descending colon. Mild altered vascularity, congestion (edema), and friability were found as patches surrounded by normal mucosa in the transverse colon, ascending colon, and cecum with associated terminal ileitis (Figure 1). The ileocecal valve was stenotic and difficult to intubate. The limited visualized portion appeared normal. Biopsy revealed mild chronic active colitis negative for granulomas and dysplasia (Figure 2).

 

**Figure 3:**
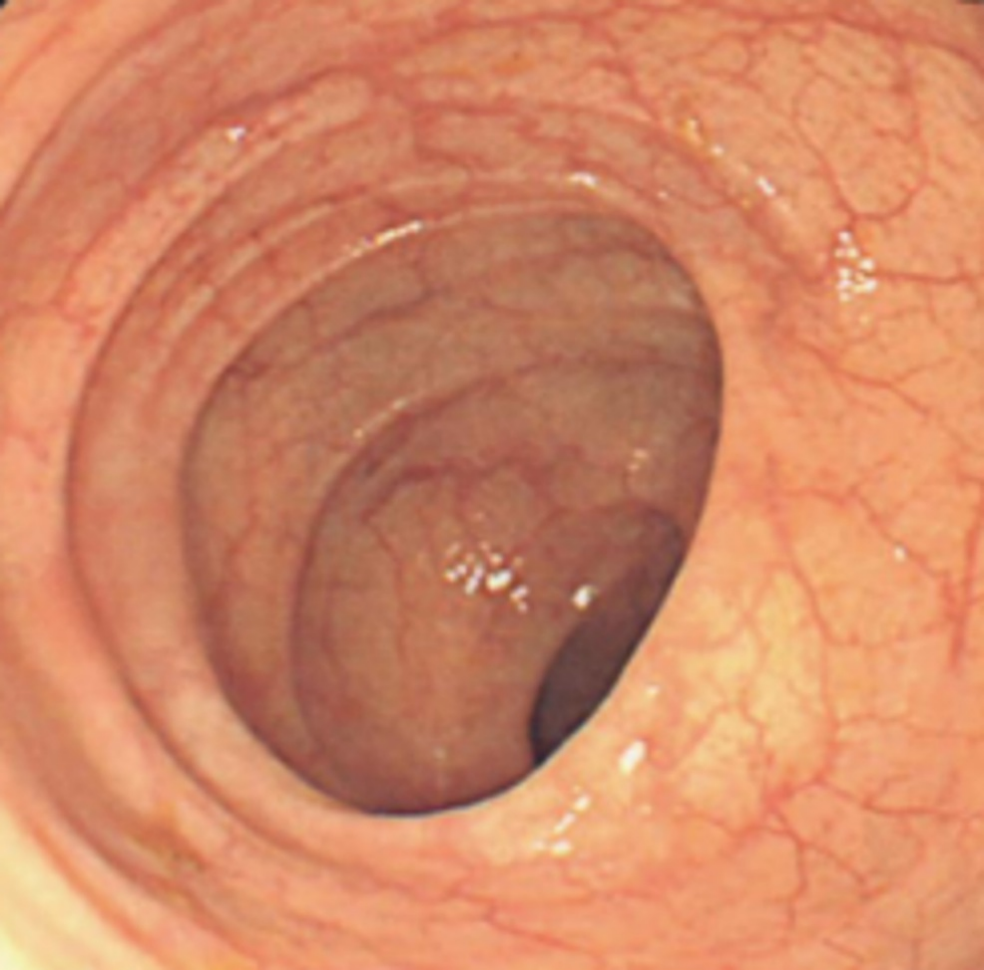
Endoscopic findings after treatment with steroids and IV adalimumab show benign mucosa

**Figure 4:**
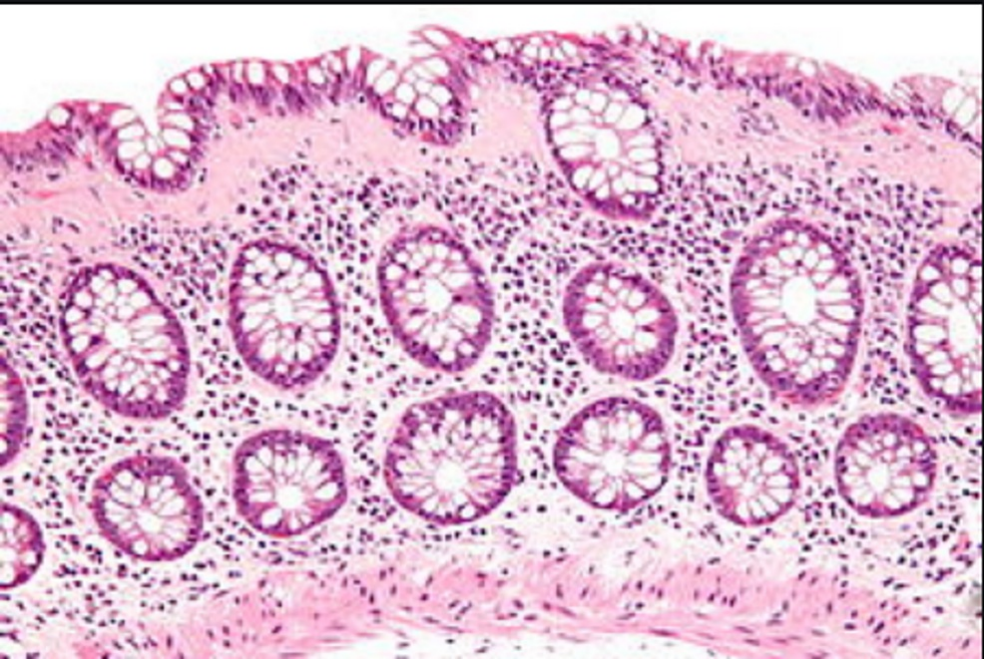
Hematoxylin and eosin stain biopsy exhibits findings of increased subepithelial collagen, consistent with collagenous colitis

CT enterography showed narrowing of the terminal ileum with focal wall thickening and two more strictures in the small bowel. The patient was diagnosed with CD, started on IV steroids, and improved significantly. She was eventually transitioned to adalimumab outpatient. Three months later, she was readmitted with 10-day non-bloody diarrhea despite being compliant with adalimumab. After ruling out infections, including *Clostridium difficile*, she was started on oral steroids. Colonoscopy showed improved colitis from previous examination (Figure 3) but biopsy revealed increased subepithelial collagen plate with minimal mucosal distortion suggestive of CC (Figure 4).

She was started on budesonide in addition to adalimumab with no improvement. Review of medications showed selective serotonin reuptake inhibitor usage, which was discontinued. She was counseled to quit smoking but unsuccessful. Off the oral steroids, she continued to have 10-15 bowel movements. Trials of cholestyramine, bismuth, and loperamide were unsuccessful. Repeat colonoscopy showed endoscopically benign mucosa but continued presence of CC on biopsy. Given the refractoriness of symptoms, the patient was referred to a tertiary IBD center.

## Discussion

MC is a clinicopathologic disorder, histologically subdivided into LC with increased intraepithelial lymphocytes (>20/100 colonic surface epithelial cells) and CC with atypically thickened subepithelial collagen band >10 μm [4]. MC is postulated to develop via an immunological response to various mucosal insults in predisposed patients [3]. LC, in particular, is associated with other autoimmune conditions such as celiac disease, psoriasis, and rheumatoid arthritis [5]. 

IBD manifests as endoscopically visible ulcerated mucosa, loss of vascularity, edema, friability, strictures, and so on. UC usually presents as bloody diarrhea while CD presents as fistulas, obstruction, and abdominal masses. Our patient initially presented with diarrhea, elevated inflammatory markers, and endoscopic and imaging findings consistent with CD. She was appropriately treated with IV steroids and adalimumab. Recurrence of her diarrhea prompted repeat colonoscopy, which revealed features of CC.

The relationship between IBD and MC is uncertain. MC could be a continuum of IBD; new-onset MC diagnosis was found after a median (range) of 20 (2-52) years in established IBD patients [1,5]. Conversely, established MC patients may also develop IBD, at a 17-fold increase in relative risk [6]. Additionally, cases of IBD patients in remission showed CC on biopsies [7]. Some MC patients reported a family history of UC [8], alluding to the role of genetic factors between the two.

Pathophysiologically, in MC, it is theorized that tumor necrosis factor-α (TNF-α) triggers the downregulation of tight junction proteins, claudin-4, -5, -8, increasing permeability, thus leading to diarrhea [9]. Permeability is also increased by non-steroidal anti-inflammatory drugs and proton-pump inhibitors, common MC triggers, that allow antigens to pass into the lamina propria provoking lymphocytosis [2]. Increased expression of TNF-α, interferon-γ, and the Th1-specific transcription factor T-BET in patients with MC who eventually developed IBD suggests a common immunological pathophysiological pathway in the MC-to-IBD transformation [3,10]. Few studies found associations between human leukocyte antigen haplotypes, DQ2 and DQ8, and risk of CC, LC, and IBD development [11-15]. Gut dysbiosis has been noted in both IBD and MC, again suggesting a common pathophysiological pathway [16-18]. The association between smoking and IBD is well established. Recent meta-analysis demonstrates that active smoking significantly increased incidence of MC with OR 3.58 [19].

Early consideration and diagnosis of MC in patients with IBD is important in tailoring the treatment regimen. Primary recommendation is cessation of potential triggers. For milder disease, cholestyramine, atropine/diphenoxylate, bismuth subsalicylate, and loperamide may be utilized. For moderate-to-severe disease, budesonide is recommended. Refractory patients require additional medications, such as infliximab, azathioprine, and methotrexate. Likewise, anti-TNF-α agents have demonstrated a good response rate [20]. The most severe cases, like poorly controlled IBD cases, require surgical diversion, colectomy, and diverting ostomy.

IBD has known complications such as malignancy and immune-mediated diseases such as ankylosing spondylitis and autoimmune thyroid disease. A systematic review showed an association between rheumatic diseases and MC [4,5], but no real conclusions can be made regarding MC and its association with classic IBD complications [4]. The complication rates and prognosis with MC-IBD overlap are unknown.

## Conclusions

Our case highlights the possibility of development or co-existence of CC in a patient with CD. Whether MC is a spectrum of IBD or it is exacerbated by trigger factors is unclear. MC should be considered as a differential in IBD patients who were previously controlled or not responding to appropriate therapy presenting with chronic watery diarrhea. Prompt diagnosis can help early management of the disease and thereby improve quality of life for the patients and reduce healthcare costs.
